# Annual acknowledgement of reviewers

**DOI:** 10.1186/s12881-016-0277-3

**Published:** 2016-02-12

**Authors:** Timothy R. Sands

**Affiliations:** BioMed Central, Floor 6, 236 Gray’s Inn Road, London, WC1X 8HB, UK

## Abstract

The editors of BMC Medical Genetics would like to thank all our reviewers who have contributed to the journal in Volume 16 (2015).

**Aiysha Abid**

Pakistan

**Gijs Afink**

Netherlands

**Jose Agundez**

Spain

**Shafqat Ahmad**

Sweden

**Waqas Ahmed**

Pakistan

**Arvind Ahuja**

India

**Loubna Akhabir**

Canada

**Alain Fischer**

France

**Marielle Alders**

Netherlands

**Thaddeus Allen**

USA

**Prabha Andraweera**

Sri Lanka

**Kübler Andrzej**

Poland

**Paolo Aretini**

Italy

**Dan Arking**

USA

**Tahir Atik**

Turkey

**Kit Sing Au**

USA

**Ruxandra Bachmann-Gagescu**

Switzerland

**Guney Bademci**

USA

**Jeehyeon Bae**

South Korea

**Deeksha Bali**

USA

**Yoshiyuki Ban**

Japan

**Siddharth Banka**

UK

**Anu Bashamboo**

France

**Sulman Basit**

Saudi Arabia

**Claiton Bau**

Brazil

**Olivier Becherel**

Australia

**Jirair Bedoyan**

USA

**Petra Bergström**

Sweden

**Dwaipayan Bharadwaj**

India

**Jocelyn Biagini Myers**

USA

**Silvia Bione**

Italy

**Douglas Bittel**

USA

**Alan Bittles**

Australia

**Nevado Blanco**

Spain

**Alessandra Boletta**

Italy

**Hanno Bolz**

Germany

**Guntram Borck**

Germany

**Yvonne Böttcher**

Germany

**Nabila Bouatia-Naji**

France

**Patrice Bouvagnet**

France

**Kari Branham**

USA

**Hilgo Bruining**

Netherlands

**Nicola Brunetti-Pierri**

Italy

**Alfredo Brusco**

Italy

**Maria Bruzelius**

Finland

**Camron Bryant**

USA

**Dejan Budimirovic**

USA

**Monika Buraczynska**

Poland

**Kathryn Burdon**

Australia

**Jacinta Bustamante**

France

**Jiping Cai**

China

**Vijayasarathy Camasamudram**

USA

**Xin Cao**

China

**Cynthia Cardoso**

Brazil

**John Carulli**

USA

**Aline Carvalho**

Brazil

**Stéphane Cauchi**

France

**Alvaro Cerda**

Brazil

**Zafer Çetin**

Turkey

**Serdar Ceylaner**

Turkey

**Hyojin Chae**

South Korea

**Imen Chamkha**

Tunisia

**Sally Chappell**

UK

**Dongbao Chen**

USA

**Edwin Chen**

UK

**Lijia Chen**

Hong Kong

**Sam Cheng**

USA

**Yu Kyung Cho**

South Korea

**Murim Choi**

South Korea

**Jan Christian**

USA

**Paraskevi Christofidou**

UK

**Denise Christofolini**

Brazil

**Mete Civelek**

USA

**Annique Claringbould**

Netherlands

**Bernard Cohen**

USA

**Andrew Collins**

Norway

**Martina Cornel**

Netherlands

**Fernanda Correa**

Brazil

**Laura Crisponi**

Italy

**Katalin Csiszar**

USA

**Ivon Cuscó**

Spain

**Aparecido Da Cruz**

Brazil

**Soheil Dadras**

USA

**Kausik Das**

India

**Mariza De Andrade**

USA

**Carlos De Brasi**

Argentina

**Gianpaolo De Filippo**

France

**Andréia Rosane De Moura Valim**

Brazil

**Nicolas De Roux**

France

**Jean De Schepper**

Belgium

**Deepthi De Silva**

Sri Lanka

**Margaret Deangelis**

USA

**Maria Lisa Dentici**

Italy

**Terry Derks**

Netherlands

**Andrew Dewan**

USA

**Teresa Di Desidero**

Italy

**Patricia Dickson**

USA

**Giulio Disanto**

UK

**Vajira Dissanayake**

Sri Lanka

**Robert Dodd**

USA

**Ranhui Duan**

China

**Christele Dubourg**

France

**Kate Duffus**

UK

**Abhijit Dutta**

India

**Thomas Ebert**

Germany

**Todd Edwards**

USA

**Sherif El-Khamisy**

UK

**Deborah Elstein**

Israel

**Barry Eng**

Canada

**Ayelet Erez**

Israel

**Pascal Escher**

Switzerland

**Stephen Eyre**

UK

**Walid Fakhouri**

USA

**Tove Fall**

Sweden

**David Fardo**

USA

**Sanja Farkas**

Sweden

**Agustin Fernandez**

Spain

**Jose Fernandez**

USA

**Massimiliano Ferrara**

Italy

**Marco Fichera**

Italy

**Karla Figueroa**

USA

**Alarcon Flora**

France

**Sandra Founds**

USA

**Kevin Foust**

USA

**Lude Franke**

Netherlands

**Judith Fridovich-Keil**

USA

**Ricardo Fujita**

Peru

**Nobuo Fuse**

Japan

**Marta Futema**

UK

**Suzanne Gage**

UK

**Daniel Gale**

UK

**Miguel Garcia-Gonzalez**

Spain

**Elena Garcia-Martin**

Spain

**Scott Garman**

USA

**Tuoyu Geng**

China

**Reza Ghassemifar**

Australia

**Michael Gill**

Ireland

**Roberto Giugliani**

Brazil

**Johannes Gjerstad**

Norway

**Christos Gkogkas**

UK

**Paul Goodfellow**

USA

**Ajit Gorakshakar**

India

**Steven Graves**

USA

**Annette Grueters**

Germany

**Ewa Grzybowska**

Poland

**Jordi Guimera**

Germany

**Alistair Gunn**

New Zealand

**Tingwei Guo**

USA

**Yiran Guo**

USA

**Dongchuan Guo**

USA

**Patricia Hall**

USA

**Ian Hastings**

UK

**Ziarih Hawi**

Australia

**Peter Henneman**

Netherlands

**Imma Hernan**

Spain

**Stephanie Hicks**

USA

**Alan Hinnebusch**

USA

**Anke Hinney**

Germany

**Rosario Hirata**

Brazil

**Kim Hirshfield**

USA

**Mads Vilhelm Hollegaard**

Denmark

**Amjad Horani**

USA

**Yukio Horikawa**

Japan

**H Dean Hosgood**

USA

**Xuwei Hou**

China

**Judie Ann Howrylak**

USA

**I-Chueh Huang**

USA

**Dirk Hubmacher**

USA

**Annika Hult**

Sweden

**Mahmood Hussain**

USA

**Adlette Inati**

Lebanon

**Nobuhisa Ishikawa**

Japan

**Naoko Iwasaki**

Japan

**Francis Jacob**

Switzerland

**Michelle Jacobs**

USA

**Henning Jansen**

Germany

**Monika Jasek**

Poland

**Juraj Javor**

Slovakia

**Young Joo Jeon**

South Korea

**Andrew Jheon**

USA

**Chunyan Ji**

China

**Nathalie Jonca**

France

**Rebecca Jones**

UK

**Paweł Jóźków**

Poland

**Kerstin Junker**

Germany

**Alexander Kanapin**

UK

**Chitra Kannabiran**

India

**Stavroula Kanoni**

UK

**Chuan-Liang Kao**

Taiwan

**Muthusamy Karthikeya**

India

**Catherine Keegan**

USA

**Kim Keeling**

USA

**Jonathan Kerr**

Colombia

**Daniel Kim**

USA

**Tomas Kirchhoff**

USA

**Dimitra Kiritsi**

Germany

**Robert Klein**

USA

**Kleopas Kleopa**

Cyprus

**Stian Knappskog**

Norway

**Jeroen Knijnenburg**

Netherlands

**Yuta Kochi**

Japan

**Daisuke Kohno**

Japan

**Inke König**

Germany

**Fariba Koohdani**

Iran

**Neeltje Kootstra**

Netherlands

**Dieter Kotzot**

Austria

**Kenneth Kraemer**

USA

**Michael Kruer**

USA

**Ho-Chang Kuo**

Taiwan

**Diederik Kuster**

Netherlands

**Baiba Lace**

Latvia

**Joseph Lam**

Canada

**Christina Lampe**

Germany

**Pablo Lapunzina**

Spain

**Damien Lederer**

Belgium

**Jong Keuk Lee**

South Korea

**Sang Hong Lee**

Australia

**Robert Lee**

USA

**Paola Leone**

USA

**Elizabeth Leslie**

USA

**Ariadne Letra**

USA

**Andrew Levine**

USA

**Keshen Li**

China

**Qiaoli Li**

USA

**Peining Li**

USA

**Elaine Lim**

USA

**Xu Lin**

China

**Ying-Ju Lin**

Taiwan

**Anna Lindstrand**

Sweden

**Beata Lipska**

Poland

**Zehuan Liu**

China

**Yutao Liu**

USA

**Javier Llorca**

Spain

**Grisel Lopez**

USA

**Karen Lower**

Australia

**Weining Lu**

USA

**Zhengfei Lu**

USA

**Emanuela Lucci-Cordisco**

Italy

**Helen Lunt**

New Zealand

**Ma Luo**

Canada

**Donia Macartney-Coxson**

New Zealand

**Deborah Mackay**

UK

**Kamlesh Madan**

Netherlands

**Irene Madrigal**

Spain

**Sajid Malik**

Pakistan

**Andigoni Malousi**

Greece

**Francesca Malvestiti**

Italy

**Eirini Manoli**

USA

**Forbes Manson**

UK

**Sahar Mansour**

UK

**Giuseppe Marangi**

Italy

**Asaf Marco**

Israel

**Donna Martin**

USA

**Flavia Martinez**

USA

**Kieren Mather**

USA

**Koichi Matsuda**

Japan

**Inas Mazen**

Egypt

**Kenneth Mcelreavey**

France

**Sean Mcghee**

USA

**Jim Mcgill**

Australia

**Heather Mclaughlin**

USA

**Giuseppe Merla**

Italy

**David Meyre**

France

**Adam Milam**

USA

**Mirta Milic**

Croatia

**Martina Minnerop**

Germany

**Ryan Minster**

USA

**Monica Miozzo**

Italy

**Mª Dolores Miramar**

Spain

**Kenichiro Miura**

Japan

**Rikke Møller**

Denmark

**David Monk**

Spain

**Adriana Montaño**

USA

**Sam Moore**

South Africa

**Ana Morales**

USA

**Rinki Murphy**

New Zealand

**Balint Nagy**

Hungary

**Shiva Nasirabadi**

USA

**Sonia Navas-Martin**

USA

**Mark Nellist**

Netherlands

**Georges Nemer**

Lebanon

**Giovanni Neri**

Italy

**Cornelis Noordam**

Netherlands

**Antonio Novelli**

USA

**Małgorzata Nowaczyk**

Canada

**Michael O'Callaghan**

Australia

**Tsutomo Ogata**

Japan

**Gisela Orozco**

UK

**Harry Ostrer**

USA

**Olivier Outteryck**

France

**Darius Paduch**

USA

**Edenir Palmero**

Brazil

**John Pappas**

USA

**Silvia Paracchini**

UK

**Rossella Parini**

Italy

**Jason Park**

USA

**Ji Wan Park**

South Korea

**David Parry**

UK

**Karen Pawlowski**

USA

**Daniel Petrovic**

Slovenia

**Massimo Pifferi**

Italy

**Renato Polimanti**

USA

**Michael Portman**

USA

**Renata Posmyk**

Poland

**David Potter**

USA

**Megan Puckelwartz**

USA

**Zilong Qiu**

China

**Hui-Qi Qu**

USA

**Singh Rajender**

India

**Feliciano Ramos**

Spain

**Ahmed Rebai**

Tunisia

**Hudson Reddon**

Canada

**Heiko Reutter**

Germany

**Stefano Romeo**

Sweden

**Nurit Rosenberg**

Israel

**Estela Rubio-Gozalbo**

Netherlands

**Víctor Ruiz-Pérez**

Spain

**Eric Rush**

USA

**Kelli Ryckman**

USA

**Maria Sabater-Lleal**

Finland

**Norihisa Saeki**

Japan

**Jørn Sagen**

Norway

**Jitendra Sahu**

India

**Lyubov Salnikova**

Russia

**Ettore Salsano**

Italy

**Andrew Sandford**

Canada

**Filippo Santorelli**

Italy

**Adalberto Santos**

Brazil

**Vishnupriya Satti**

India

**Kapaettu Satyamoorthy**

India

**Ali Sazci**

Turkey

**André Scherag**

Germany

**Albert Schinzel**

Switzerland

**Benedikt Schoser**

Germany

**Margit Schraders**

Netherlands

**Valerie Schumacher**

USA

**Dominik Seelow**

Germany

**Mark Seielstad**

USA

**Wenke Seifert**

Germany

**Andrei Seluanov**

USA

**Nuno Sepulveda**

UK

**Leping Shao**

China

**Adam Shapiro**

Canada

**Aiden Shearer**

USA

**Lijing Shen**

China

**Robert Shenkar**

USA

**Jung-Bum Shin**

USA

**Yin Shugart**

USA

**Marilia Silva**

Brazil

**Louise Simard**

Canada

**Robert Sladek**

Canada

**Anne Slavotinek**

USA

**Joshua Sommers**

USA

**Pavel Soucek**

Czech Republic

**Kamna Srivastava**

India

**Sandra Staffieri**

Australia

**Joshua Starmer**

USA

**Lois Starr**

USA

**Joshua Stern**

USA

**Giovanni Stevanin**

France

**Elsdon Storey**

Australia

**John Streicher**

USA

**Ken Sugimoto**

Japan

**Bhoom Suktitipat**

Thailand

**Guang Sun**

Canada

**Isaac Sundar**

USA

**Miroslav Svoboda**

Czech Republic

**Gote Swedberg**

Sweden

**Julia Szendrödi**

Germany

**Hiromi Tanaka**

USA

**Clara Tang**

Hong Kong

**Yaohui Tang**

USA

**Kai-Fu Tang**

China

**Nelson Tang**

Hong Kong

**Marco Tartaglia**

Italy

**Nicole Tartaglia**

USA

**Andrew Taylor**

USA

**Bamidele Tayo**

USA

**Isabel Tejada Minguez**

Spain

**Frédérique Tesson**

Canada

**Sophie Tezenas Du Montcel**

France

**Sebastien Theriault**

Canada

**Christian Thiel**

Germany

**Xiao-Li Tian**

China

**Maarit Tiirikainen**

USA

**Eduardo Tizzano**

Spain

**Janettet Tong**

Australia

**Edina Torgyekes**

USA

**Jorg Tost**

France

**Lisbeth Tranebjærg**

Denmark

**Chang-Yong Tsao**

USA

**Sebahat Turgut**

Turkey

**Joseph Usset**

USA

**Diana Valverde**

Spain

**Marie van Dijk**

Netherlands

**Conny van Ravenswaaij**

Netherlands

**Avinash Veerappa**

India

**Hannah Verdin**

Belgium

**Wim Vermeulen**

Netherlands

**Alexandre Vieira**

USA

**Robert Vierkant**

USA

**Saroja Voruganti**

USA

**Konstantinos Voskarides**

Cyprus

**Lahcen Wakrim**

Morocco

**Keith Walley**

Canada

**Yu Wang**

Singapore

**Naveed Wasif**

Pakistan

**David Watkins**

Canada

**Ronald Wek**

USA

**Sven Wenske**

USA

**Michael Weston**

USA

**Marquitta White**

USA

**Christina Willenborg**

Germany

**Esko Wiltshire**

New Zealand

**Nicole Wolf**

Netherlands

**Henry Wood**

UK

**Lawrence Shih Hsin Wu**

Taiwan

**Jia-Feng Wu**

Taiwan

**Yuliang Wu**

Canada

**Qiang Wu**

Singapore

**Ying Wu**

USA

**Junxia Xie**

China

**Kazuya Yamagata**

Japan

**Toshiyuki Yamamoto**

Japan

**Liu Yangyang**

China

**Svetlana Yatsenko**

USA

**Yuanqing Ye**

USA

**Ashwini Yenamandra**

USA

**Fuming Yi**

USA

**Terry-Lynn Young**

Canada

**Jacob Yount**

USA

**Anna Zampetaki**

UK

**Tetyana Zayats**

Norway

**Qi Zeng**

USA

**Juan Carlos Zenteno**

Mexico

**Jin-An Zhang**

China

**Mingzhi Zhang**

China

**Haiying Zhang**

China

**Minwen Zhou**

China

**Marcella Zollino**

Italy

**Orsetta Zuffardi**

Italy

**Nathan Zwagerman**

USA

**Christiane Zweier**

Germany

